# Conceptualizing care partners' burden, stress, and support for reintegrating Veterans: a mixed methods study

**DOI:** 10.3389/fpubh.2023.1295627

**Published:** 2024-02-19

**Authors:** Nicholas A. Rattray, Mindy Flanagan, Allison Mann, Leah Danson, Ai-Nghia Do, Diana Natividad, Katrina Spontak, Gala True

**Affiliations:** ^1^VA Health Services Research and Development Center for Health Information and Communication, Roudebush Veteran Affairs Medical Center, Indianapolis, IN, United States; ^2^Regenstrief Institute, Inc., Indianapolis, IN, United States; ^3^Department of General Internal Medicine and Geriatrics, Indiana School of Medicine, Indianapolis, IN, United States; ^4^Department of Psychological Services, University of Indianapolis, Indianapolis, IN, United States; ^5^South Central Mental Illness Research, Education, and Clinical Center, Southeast Louisiana Veterans Health Care System, New Orleans, LA, United States; ^6^Section of Community and Population Medicine, Louisiana State University School of Medicine, New Orleans, LA, United States

**Keywords:** military Veterans, caregiver burden, caregiving, mixed methods, health services, veteran reintegration

## Abstract

**Background:**

People who support Veterans as they transition from their military service into civilian life may be at an increased risk of psychological distress. Existing studies focus primarily on paid family caregivers, but few studies include spouses and informal non-family “care partners.” We sought to identify key challenges faced by care partners of Veterans with invisible injuries.

**Methods:**

Semi-structured interviews were conducted with 36 individuals involved in supporting a recently separated US military Veteran enrolled in a 2-year longitudinal study. CPs completed validated measures on perceived stress, caregiving burden, quality of their relationship, life satisfaction, and flourishing. Independent *t*-tests were used to compare cases in these groups on caregiving burden, quality of their relationship, life satisfaction, and flourishing. Care partners were categorized as reporting high and low levels of stress. Exemplar cases were used to demonstrate divergences in the experiences of CPs with different levels of stress over time.

**Results:**

Care partners reported shifts in self-perception that occurred from supporting a Veteran, emphasizing how they helped Veterans navigate health systems and the processes of disclosing health and personal information in civilian contexts. Exemplar cases with high and low burdens demonstrated divergent experiences in self-perception, managing multi-faceted strain, and coping with stress over time. Case studies of specific care partners illustrate how multi-faceted strain shifted over time and is affected by additional burdens from childcare, financial responsibilities, or lack of education on mental health issues.

**Conclusions:**

Findings suggest the unique needs of individuals who support military Veterans with invisible injuries, highlighting variations and diachronic elements of caregiving. This sample is younger than the typical caregiver sample with implications for how best to support unpaid care partners caring for Veterans in the early to mid-period of their use of VA and civilian health services.

## Introduction

Since 2001, there has been increased focus on the “invisible wounds of war,” which refers to mental health issues and cognitive impairments resulting from military service in the twenty-first century (1). Longer deployments as well as advances in combat medicine (2), have led a significant proportion of returning soldiers to report high levels of psychological and physical distress (3), and difficulties in reintegrating into civilian society (4–6). Compared to past eras, there is greater emphasis on diagnosing Post Traumatic Stress Disorder (PTSD), traumatic brain injuries, and depressive symptoms in post-9/11 Veterans. However, the effects of these conditions are still poorly understood compared to injuries categorized as physical wounds. The medical costs for invisible conditions alone are ~$2–3 billion per year (7), and families bear a significant “private burden” of uncompensated costs related to service-connected disabilities (8).

The effects of caregiving by family members are well-established in medical literature but are largely based on geriatric populations (9). Caregivers for military and Veteran populations are more likely to be younger, have dependents, and face longer periods of care for individuals with higher disability burdens (10). For Veterans enrolled in health care with the US Department of Veterans Affairs (VA), studies have suggested that caregivers who support Veterans have higher depression and burden compared to civilian counterparts and more financial strain over time (11). This mirrors wider findings that caregiving can lead to negative impacts on the health, wellbeing, economic security, and careers of family caregivers (12–14).

Within the literature on people who are caregivers for Veterans, studies tend to focus on caregivers for military servicemembers (15, 16), enrollees in the paid VA caregiver program (17), or caregivers who support patients with a specific diagnostic condition (e.g., cancer, diabetes, etc.). Abraham et al. have demonstrated less visible forms of “emotional work” of Veteran caregivers (18), while others have examined how caregivers enrolled in VA support programs perceive their uncompensated work (19). In the wake of the two million US troops that were deployed to Iraq and Afghanistan, researchers called for more treatments focused on Veterans and their whole support system. These include family-centered interventions that account for caregiver burden and deal with psychological distress experienced by Veterans and their families (20), with greater attention paid to the early period of post-deployment adjustment (21) and the effects on spouses and children (22).

Caregivers of Veterans, specifically Veterans with invisible injuries, have unique sets of needs (23). Due to the complex nature of invisible injuries, these Veterans' care requires increased effort from caregivers (22) and associated burden (24). Previous research revealed that family and social resources available for caregivers of Veterans aid in mitigating their overall psychological distress (25). Studies have demonstrated the unique consequences of PTSD on family functioning, such as emotional numbing and withdrawal (26), how spouses are often responsible for maintaining normalcy (27), and lower levels of life satisfaction (28). Many Veteran caregivers' needs, such as emotional support, understanding Veterans' benefits, locating Veteran services, and more, go unfulfilled (16).

Research partnerships in recent years have been developed to establish a clear research agenda for US military and Veteran caregivers based on existing studies from RAND and efforts from the Elizabeth Dole Foundation (29). Extant studies tend to focus on paid caregivers in the VHA Program of Comprehensive Assistance for Family Caregivers (PCAFC), leaving gaps in knowledge about unpaid caregivers and Veterans who are not enrolled in VHA health care.

Direct engagement with military and Veteran caregivers has contributed to the development of the “Military and Veteran Caregiver Experience Map,” a conceptual model designating stages in caregivers' “journeys” (30). More recent research has focused on suicidal ideation among military caregivers (31) and on evaluating existing programs, such as a study showing that a VA caregiver program was effective in reducing anxiety, depression, caregiver burden, and overall stress (32).

In this study, we adopted the term “care partner,” (CP) which aligns with recent calls for an inclusive approach to caregiving that recognizes the wide range of activities and roles individuals may perform in support of those they care for (33, 34). In addition to spouses, this term enabled us to recruit friends, siblings, parents, or others who might be nominated by Veteran study participants. Although CPs play a significant role in the reintegration processes of post-9/11 Veterans with invisible injuries, less is known about how they subjectively view the burden and stress of caregiving, as well as how it impacts their wellbeing. More specifically, we build on an existing conceptual definition of burden (35) to incorporate the distinct perspectives of CPs for military Veterans. This conceptualization of caregiver burden incorporates the following three attributes: self-perception (perceived negative and positive feelings or aspects related to the caregiving role), multifaceted strain (multiple types of strain associated with caregiving, such as health problems, psychological stress, social isolation, or financial problems), and time (change in caregiving burden over time). Incorporating temporality from repeated interviews and analysis of personal history aligns with a life course perspective attentive to continuity and provides context to change in caregivers' experiences (36, 37) and shifts common in Veterans' lives (38). We adopt a mixed methods approach to understand why some caregivers report greater strain and burden and how their narratives diverge.

This study examines the role that informal CPs play in the lives of military Veterans with invisible injuries (mental or cognitive health conditions) in the early phase of their adjustment to civilian life. We do so by using a mixture of quantitative and qualitative methods to offer a more comprehensive view of caregiving and discover potential ways to assist Veteran CPs. The specific aims are to (1) examine associations between CP characteristics and outcomes (flourishing, stress, burden); (2) to describe CP perspectives on how they support the Veteran in their life; and (3) through mixed method analysis, to understand patterns of convergence or divergence between CPs reporting high and low levels of burden.

## Methods

### Design

We adopted a mixed methods approach to offer breadth and depth of understanding beyond what qualitative or quantitative methods would allow alone (39). Specifically, our mixed method design was an explanatory unidirectional approach (40) where questionnaire data merged with qualitative findings from thematic analysis and in-depth case study analysis. Interview data was reported using a qualitative descriptive design with narratives (41). The study was approved by the Indiana University Institutional Review Board and VA Research and Development Committee.

### Participants

This study included a sample of CPs participating in a 2-year longitudinal study that examined community reintegration among military Veterans with an invisible injury (42), which includes clinical diagnosis of post-traumatic stress disorder, depression, anxiety, traumatic brain injury, or other mental or cognitive health condition and their care partners. As reported elsewhere, 91% of these Veteran participants had a disability rating (43). A CP is defined by the Veteran as someone who supports him/her in an area considered important to reintegration (family/social life, school, work, rehabilitation, etc.), typically a family member, partner, friend, or neighbor (33). Following the baseline visit, Veterans were encouraged to nominate an individual who currently supports their adjustment to civilian life. Nominated individuals were contacted via phone to describe the study and inquire about their interest in study participation. Of the 75 Veterans, 48 nominated a CP, which led to a convenience sample of 36 CPs enrolled in the study.

### Procedures

Before collecting any data, a member of the research team discussed the study aims with CPs and obtained consent and HIPAA authorization. Data collection included a mix of quantitative and qualitative data. The qualitative data collection involved semi-structured open-ended interviews. CPs received a $25 gift card for each assessment. The quantitative data collection included demographic information and self-administered close-ended questionnaires.

#### Semi-structured interviews

Following a semi-structured interview guide, CPs were asked about their role in supporting the Veterans and how the Veteran's reintegration impacts their own health and wellbeing. Specific topics that were covered included the CP's role in the Veteran's transition experience, family or social issues, financial/economic issues, and their perceptions on the overall reintegration experience. CPs were interviewed at baseline, 12 months, and 24 months and each interview lasted 60–90 min. Interviews were conducted individually and were either face-to-face in a private room or virtually by phone or videoconferencing, depending upon participant preference and in accordance with protocols adopted during the COVID-19 pandemic period. The digitally recorded interviews were transcribed verbatim, checked for accuracy by listening to the tapes and comparing them with the transcripts, and de-identified.

### Measures

#### Caregiving burden

The Zarit Burden Interview (ZBI) assesses the perceived burden of caregiving (i.e., the extent to which caregiving causes stress, and interferes with the caregiver's health and other relationships or responsibilities). This 12-item questionnaire includes a 5-point Likert scale (0 = Never, 4 = Nearly always). Summed scores range from 0 to 48, such that a higher perceived burden is indicated by higher scores (44). The cutoff between low and high burden has been reported as 12 (45), 13 (46), and 19 (47). A high burden was defined as 13 points and higher for this study. Studies have suggested that the caregiving burden is multi-dimensional, including role strain (how caregiving conflicts with other roles) and personal strain (individualized stress) (48, 49).

#### Perceived stress

The Perceived Stress Scale (PSS) is a 10-item measure that assesses stress on a 0 (Never) to 4 (Very Often) scale (50). Items include “In the last month, how often have you felt that you were unable to control the important things in your life?” and “In the last month, how often have you felt difficulties were piling up so high that you could not overcome them?” Scores range from 0 to 40 where normative scores are 12.1 (SD = 5.9) for men and 13.7 (SD = 6.6) for women. For this study, high stress was operationalized as a score of 14 or higher.

#### Flourishing

The “Flourishing measure” includes six domains that contribute to sustained wellbeing (51) and has been validated in a cross-cultural study (52). The Flourishing measure includes the following six domains: happiness, health (mental and physical), meaning and purpose, character, social relationships, and financial stability, an enabler to the other five domains. The response set ranges from 1 (Strongly disagree) to 7 (Strongly agree). The Secure Flourishing Index (SFI) is an average of all 12 items, where higher scores indicate higher levels of flourishing.

#### Life satisfaction

The Satisfaction with Life Scale (SWLS) assesses an individual's quality of life as they experience and evaluate it. When completing the SWLS, respondents indicate the extent to which they agree or disagree with five items (1 = Extremely dissatisfied, 7 = Highly satisfied) (53). The SWLS has good validity and temporal stability (54). Scores are summed for the five items. Extreme dissatisfaction is operationalized as a score in the 5–9 range, neutral satisfaction is a score of 20, and extreme satisfaction is a score in the 31–35 range.

#### Quality of relationship

The Quality of Relationship Scale assesses the CP's relationship with the Veteran, which is adapted from the mutual communal relationship scale (55–57). This scale has 10 items with responses that range from 1 (Never) to 4 (Always), with five questions focused on the giving aspect and five questions on the receiving aspects of the relationship (58).

#### Caregiver resources

A list of 14 resources available to CPs was included to determine the extent to which CPs access these resources. Based on previous studies (59), this list of resources included informal sources of information, religious networks, health care, mental health care, wellness activities, financial support, case management, and support groups. Respondents indicated whether they had used the resource and the extent to which it was helpful (1 = “Not at all,” 2 = “Somewhat,” 3 = “Very”).

### Data analysis procedures

**Figure 1:**
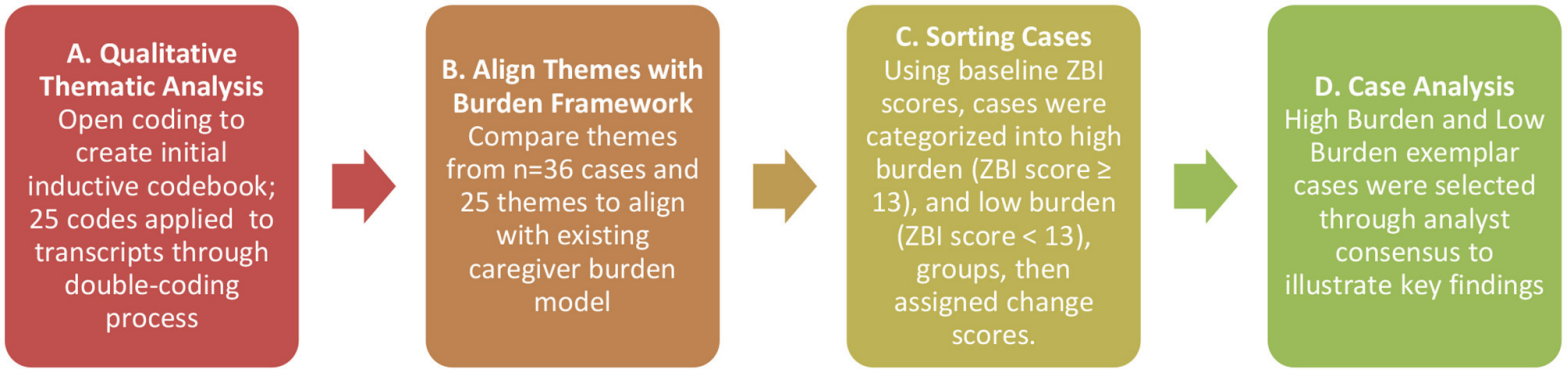
Data analysis process.

The overall mixed methods approach is described in Figure 1. First, a qualitative thematic analysis combined with matrix analysis (60) was undertaken to develop cross-cutting patterns from interviews with CPs. Three coders independently read interview transcripts in “open coding,” where each analyst inductively identified relevant excerpts with a provisional label (61). Analytic memos were written regularly during open coding to connect emergent content related to community reintegration. Case summaries were compared using a data matrix to further identify themes and achieve data saturation. A codebook was developed and refined until a shared understanding was achieved. Next, at least two team members independently coded each transcript; pairs met in person to review the double-coded transcripts and resolve discrepancies through consensus. Qualitative data was coded and analyzed using NVivo (62). Subsequently, themes were aligned with concepts from the caregiver burden model (i.e., self-perception, multi-faceted strain, disclosure, navigation, resources, needs, and strategies).

Consistent with the study aims, cases were sorted using baseline ZBI scores. Cases were categorized into high burden (ZBI score ≥ 13, 10/34, 29.4%) and low burden (ZBI score <13, 24/34, 70.6%) groups for focused analysis of interview transcripts according to their baseline burden score. Combining 12-month scores for burden with baseline burden scores, cases were categorized as staying high (4/27, 14.8%), low (17/27, 63.0%), shifting from high to low burden (4/27, 14.8%), or low to high burden (2/27, 7.4%). Seven CPs did not complete surveys at 12 months.

For the quantitative measures, descriptive statistics (e.g., mean, frequencies) were calculated to characterize the sample in terms of demographic variables and psychosocial outcomes. To examine associations between CP characteristics and outcomes, Pearson correlations were calculated among PSS, SFI, SWLS, Zarit Burden, and Quality of Relationship Scale scores. Additionally, to compare CPs reporting high burden with those reporting low burden, independent *t*-tests were used to compare these groups on PSS, SFI, SWLS, and Quality of Relationship Scale scores. To account for multiple comparisons increasing the possibility of a Type I error, a False Discovery rate adjustment was made (63).

In the next stage of analysis, baseline ZBI scores were used to categorize cases into high-burden (ZBI score ≥ 13, 10/34, 29.4%) and low-burden (ZBI score <13, 24/34, 70.6%) groups. Combining 12-month scores for burden with baseline burden scores, cases were categorized as staying high (4/27, 14.8%), low (17/27, 63.0%), shifting from high to low burden (4/27, 14.8%), or low to high burden (2/27, 7.4%). Seven CPs did not complete surveys at 12 months. In the last phase of analysis, we selected exemplar cases that illustrated cross-cutting findings and changes in burden over time.

## Results

### Sample characteristics

Of the 36 CPs, a majority were women (72.2%) and married or partners (72.2%) to the Veteran for whom they provided care (see Table 1). Consequently, most CPs lived with the Veteran (63.9%) and, on average, had known the Veteran for a considerable length of time, mean = 15.1 years (SD = 10.0, range = 0.8, 34). Only 11.1% of CPs had been caregiving for the Veteran for less than a year, while 52.8% had been a caregiver for more than 5 years. Also, 58.3% of CPs were parents or guardians to a child under the age of 18 years. Over a third (36.%) reported military service. One respondent reported participation as a paid caregiver in the VHA PCAFC.

**Table 1:** Aim 2 care partner participant characteristics (*n* = 36).

Age (years), Mean (SD)	38.3 (11.3)
**Gender** (female), *n* (%)	26 (72.2%)
**Race/Ethnicity**, *n* (%)	
Black/African American	4 (11.1%)
White/Caucasian	28 (77.8%)
Asian	3 (8.3%)
American Indian or Alaskan Native	1 (2.8%)
Hispanic/Latino	2 (5.6%)
**Served in the military (past or current)**, *n* (%)	13 (36.1%)
**Employment**, *n* (%)	
Full time (35 h/wk or more)	22 (61.1%)
Part time (<35 h/wk)	3 (8.3%)
Retired/Unemployed/Student/Homemaker	11 (30.6%)
**Financial**, *n* (%)	
Comfortable	28 (77.8%)
Just enough to make ends meet	7 (19.4%)
Not enough to make ends meet	0 (0%)
Prefer to not say	1 (2.8%)
**Relationship to Veteran**, *n* (%)	
Spouse or partner	26 (72.2%)
Parent	1 (2.8%)
Sibling	1 (2.8%)
Child	2 (5.6%)
Other non-relative (ex-spouse, friend, mentor, etc.)	7 (19.4%)
**Lives with Veteran**, *n* (%)	23 (63.9%)
**Time providing regular care to Veteran**, *n* (%)	
<1 year	4 (11.1%)
1 year to <3 years	5 (13.9%)
3–5 years	4 (11.1%)
>5 years	19 (52.8%)
Unsure	4 (11.1%)

### Associations among caregiver burden, stress, flourishing, life satisfaction, and relationship quality

As expected, burden was significantly positively correlated to perceived stress (*r* = 0.50, *p* = 0.003). Additionally, burden was significantly inversely related to secure flourishing (*r* = −0.60, *p* < 0.001) and relationship quality (*r* = −0.45, *p* = 0.008). Though an inverse trend between burden and life satisfaction was observed, this correlation was non-significant (*r* = −0.26, *p* = 0.14).

Descriptive statistics are shown in Table 2 for survey measures completed by CPs. As expected, CPs with high burden had significantly higher stress (*p* = 0.02), lower secure flourishing (*p* = 0.02), and marginally significantly lower life satisfaction (*p* = 0.08) and relationship quality (*p* = 0.06).

**Table 2:** Descriptive statistics of baseline survey measures assessed in the care partner sample by burden level.

**Measure**	**Full sample**	**Low burden**	**High burden**
	**(*n* = 34)**	**(*n* = 24)**	**(*n* = 10)**
	**Mean (SD)**	**Mean (SD)**	**Mean (SD)**
Burden	9.29 (7.23)	5.46 (3.40)	18.50 (5.38)
Stress (PSS)	13.15 (6.26)	11.38 (6.02)	17.40 (4.74)
Secure flourishing (SFI)	7.88 (1.14)	8.23 (0.87)	7.04 (1.31)
Life satisfaction (SWLS)	5.32 (1.16)	5.56 (1.20)	4.78 (0.90)
Relationship quality	3.16 (0.53)	3.28 (0.44)	2.88 (0.63)

High burden = 13 and higher.

As shown in Table 3, CPs did not access many of the resources available. The most frequently accessed resources were a religious or spiritual network and informal information sources, such as websites and articles. In contrast, formal, structured programs and resources, such as loans, support groups, case managers, caregiving training, and stipends, were rarely accessed.

**Table 3:** Frequency of care partner resources accessed and perceived as helpful (*n* = 35).

**Resource**	**Accessed resource (*n*, %)**	**Helpful (*n*, %)**
A religious or spiritual network	14 (40.0%)	14 (100.0%)
Informal sources of information (i.e., magazine articles, websites such as WebMD, and informational pamphlets)	14 (40.0%)	13 (92.9%)
Structured wellness activities for yourself (i.e., classes or group activities on exercise, yoga/meditation, and healthy eating)	13 (37.1%)	12 (92.3%)
Health care resources for yourself (i.e., doctors' appointments, visits to health care facilities)	9 (25.7%)	7 (77.8%)
Psychological counseling from a trained healthcare professional for yourself (i.e., psychologist, psychiatrist, and social worker)	7 (20.0%)	6 (85.7%)
Some other resource	7 (20.0%)	7 (100.0%)
A helping hand (i.e., loans, donations, legal guidance, or housing assistance other than VA stipends or payments)	4 (11.4%)	4 (100.0%)
A referral service for finding programs to help you with your challenges helping you care for the Veteran	3 (8.6%)	3 (100.0%)
Structured support groups such as online or in-person support groups for caregivers	3 (8.6%)	3 (100.0%)
An advocate or case manager; someone to try to coordinate help for the Veteran	3 (8.6%)	3 (100.0%)
Structured education or training (i.e., in-person classes, one-on-one training, online modules, or printed workbooks to inform you about caregiving)	1 (2.9%)	1 (100.0%)
A monthly stipend or payment from the VA in exchange for the care you provide	1 (2.9%)	1 (100.0%)
Respite care/someone who provided care to the Veteran while you did other things	1 (2.9%)	0 (0.0%)
A call-in help number for family members/friends of Veterans like yourself	1 (2.9%)	1 (100.0%)

Respondents indicating that a resource was “Somewhat” or “Very” helpful was categorized as “Helpful.”

### Qualitative themes relevant to care partner burden

**Figure 2:**
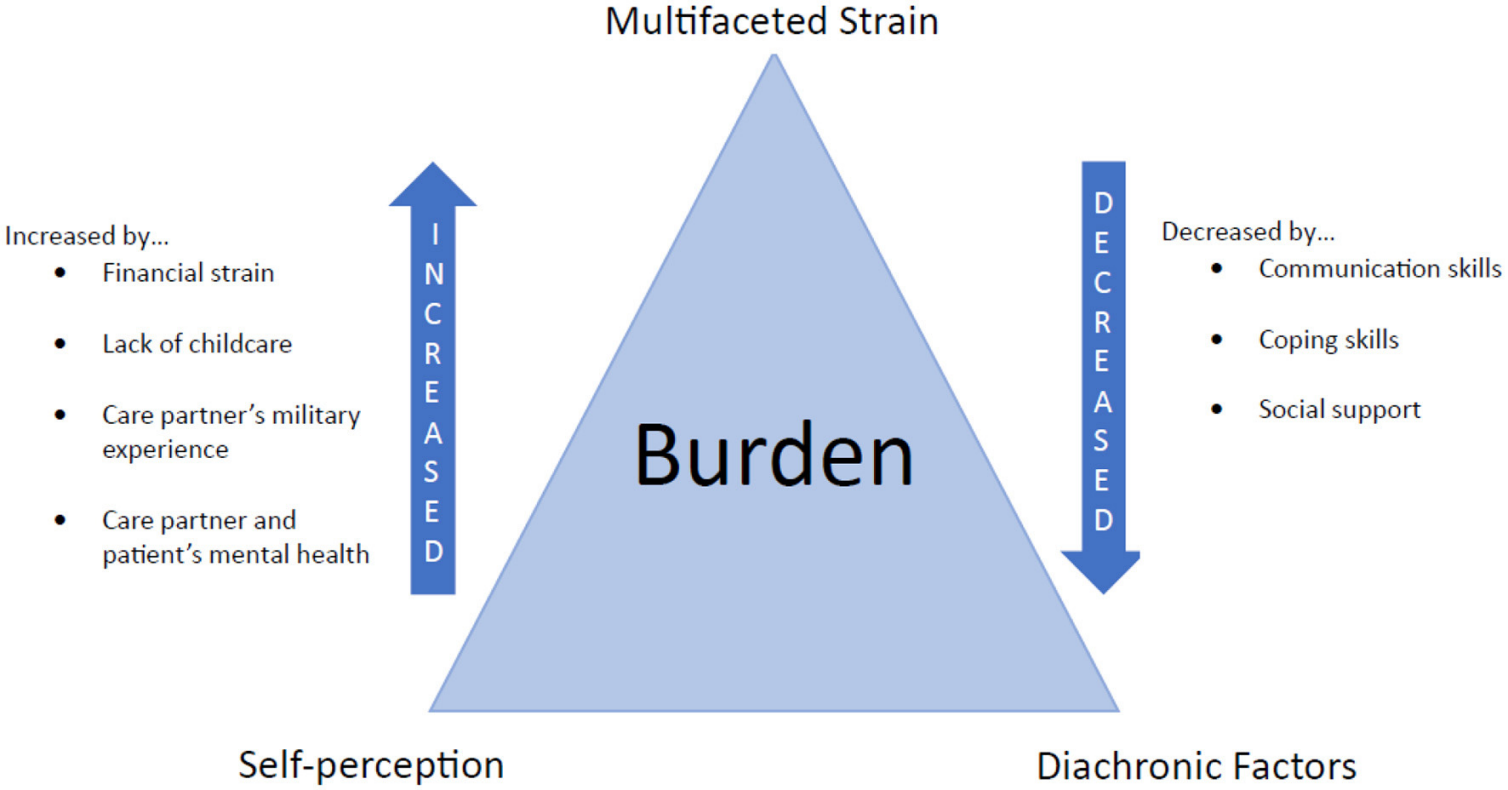
Diagram of components of care partner burden.

Based on the themes and case descriptions that follow, Figure 2 depicts how burden was conceptualized in this study. The diagram retains the dynamic relationship between self-perception, multi-faceted strain, and change over time but adds factors that increase or decrease the burdens that are specific to CPs to Veterans.

### Self-perception

CPs expressed a wide range of feelings and perceptions associated with their caregiving role. Negative emotions named included stress, anger, overwhelmed, frustrated, tired, or drained. For example, P4032 stated, “I feel like I run around like a chicken with my head cut off. So as a spouse, I feel like it's very overwhelming;” P4004 explained, “So yeah, I get stressed out. Do I know everything about what to do about everything all of the time? Absolutely not.”

However, CPs described their own qualities that were helpful in managing their caregiver role. These qualities included being adaptable, understanding the military experience, accepting their situation, being solution-focused, staying calm, and being a positive, caring person. As an example of adaptability being an asset, P4021 described, “I'm used to being flexible… it was always important to me for my husband to be happy with whatever job he's working for after the military, so if that meant we need to move a certain city, that's okay. I work in healthcare, so it's not as hard for me to find jobs.” Similarly, another CP relied on her acceptance and adaptability for managing the caregiver role. P4017 stated, “It's not a pity thing… When you sign on and you know that this is a lifestyle, you are choosing, and the choice was made that our marriage and we know that like his Marine Corps job is what's going to drive. And we were in that together. So but as a result, that means that I am not going to have a career… There is a lot of fluidity, and you have to be willing to just go with the flow and let his stuff drive it and not yours, if that makes sense.” P4006 described herself as a “fixer” meaning she will “try to figure out well, how can we fix this? Well, how can we make it better?” She viewed this as a strength in her role as a caregiver. Similarly, P4038 described herself as “a person that loves to take care of people” and “the bubbly, cheery, happy-go-lucky person. Day in and day out, will find the positive in everything.”

### Multiple responsibilities and roles

As noted by Liu et al. (30), CPs manage caregiving responsibilities alongside their other social roles. CPs reported that they helped their Veteran with daily medications, medical appointments, physical safety (i.e., falling), emotional support, and meals. One CP described monitoring her husband's mood and providing support when he appeared to need it. P4006 stated, “So at this point, that's what I really feel like that my role is really just to make sure that he has a loving support at home and that he knows that he's accepted no matter what and… recognize that oh okay, something is a little off… Either he's not communicating, or he seems a little withdrawn and then just trying to gently talk to him about are you just tired.” Similarly, when asked “Are you like worried about him?” P4012 responded, “It's kind, it's almost like a motherly worried. Because I can tell that he's anxious and that he's feeling that anxiety.” P4011 described her caregiving stress in the following way: “It can be a very, very tiring struggle sometimes to help everybody and make sure that he is still okay and still on the schedule that he needs or making it to the appointment or remembering to eat.” In addition to monitoring emotional changes, CPs reported that Veterans had angry outbursts that were hard to manage. P4020 explained: “I'm trying to keep him calm. You know… It's upsetting because he'll get angry and be upset more, which can cause the kids to get upset. Yeah, it's stressful.” Some of these CPs primarily managed household finances, daily household decisions, and household moves, or were the only driver in the household. For example, P4003 stated “So I take care of all of the financial stuff at our house.” Similarly, P4011 explained, “And I'm like the stick in the middle that controls everything (laughter). Yes. Very much so. I kind of rule everything, and I didn't like to take that spot, but I do pretty good at it (laughter).”

### Monitoring disclosure and aiding in navigation

CPs played a crucial role in supporting help-seeking behavior, including encouraging Veterans to disclose information with appropriate timing and context. P4016 explained how her husband “was so closed off” that he did not respond to initial mental health outreach, but that her presence at therapy appointments was beneficial: “I was able to kind of explain certain situations… that he maybe he didn't even realize was happening.”

CPs describe how they helped Veterans navigate through challenges related to accessing health services, scheduling appointments, and paying for health services. One CP expressed how it “was easier to navigate with two brains than one” (P4013). This CP emphasized caring for his spouse: “No, it's not a burden. I'm not even stressed either, because of the way I deal with stress, which is another thing that she is mad about, how I'm different from her on that aspect.”

P4025, a CP with high self-reported stress, described in detail attempts to help:

“I'd come home and say so when's your appointment? Oh, I couldn't do that. And so I would be encouraging and say, okay, want me to call? No, I can do it. I'll do it tomorrow. And it took the initial phone call took 5 or 6 days and then the initial appointment, there was a lot of anxiety leading up to the appointment, and the day of the appointment, there's anxiety, and so it's, which the cultural stuff?”

This CP explained the importance of persistent monitoring coupled with empathy about the Veteran's anxiety about attending scheduled appointments. Other CPs discussed how they witnessed “getting the runaround” (P4011) when trying to access services; P4004 explained that it was necessary to reach out to the Patient Advocate for assistance.

### Resources, needs, and strategies

In terms of resources that would aid CPs, respondents discussed a range of needs and strategies that they used. Several CPs suggested that focused courses or workshops delivered after military separation occurs would be beneficial with specific courses dedicated to CPs supporting Veterans. Topics that were suggested included communication skills, awareness of mental health issues, and support in navigating the VA healthcare system. P4020 suggested a coupled-focused course: “No, nobody ever contacted me and said this is what PTSD looks like—like I think that it should be mandatory for couples to go through classes before deployments and after deployments, and you know.” Other CPs expressed the need for consistency when it comes to health care services and courses to understand VA benefits, including disability and insurance from a CP perspective. P4002 proposed presentations about supporting a Veteran be held at local community centers to aid in caring efforts.

Table 3 summarizes which types of resources CPs found useful. The most accessed resources, structured wellness activities, informal information sources, and participation in spiritual or religious networks, were reported to be helpful.

### Exemplary cases

The following offer exemplar cases that illustrate high and low burdens. To demonstrate within-case changes, we likewise offer cases that remained as either high or low burden or changed their burden-level at 12 months.

#### High-burden cases

Among CPs reporting high levels of burden, P4019 was a spousal CP who himself served nearly three decades in the Navy and has a 70% service-connected disability. He left service prior to his wife, which was when they “reversed roles” and he was the primary caretaker of their children while she was deployed. He supported his wife primarily through cooking and cleaning but expressed how he wished he could provide more support for his wife but that they have communication issues. This CP scored higher for “role strain” than for “personal strain,” which converges with evidence from his interviews.

P4009 was also a Veteran who served in the Air Force for 6 years. Her husband struggled with mental health (depression, PTSD), which was a major stressor in their relationship. As a result, she felt “almost like a single parent,” caring for their four children while working as a mental health professional. This CP was categorized as having a high burden at baseline and at 12 months. She described stress from coping with the volatility of her spouse's mood, explaining how he “shuts off” and “self-isolates” from her and their children, leaving her feeling less hope for the future.

P4026 was a case where at baseline, the CP was struggling to support his girlfriend due to her own challenges with civilian reintegration, PTSD, and migraines. The CP was also a Veteran but had a smoother transition into higher education following his military service. The couple temporarily separated when the Veteran decided to move to a new state, but the CP changed his mind and moved in with her in her new residence. Though the CP had a high burden at baseline, at 12 months, the CP was categorized as low burden and had higher scores for flourishing.

#### Low burden cases

Reporting low burden, P4004 was the sole CP who had been in the VA's “Program of Comprehensive Assistance for Family Caregivers” (PCAFC) for 5 years. This CP quit her job in oral hygiene to become a full-time caregiver to her husband, a Veteran who was medically discharged and had memory problems, several physical issues, and PTSD. P4004 navigates VHA health services, taking him to multiple weekly appointments, as well as managing medication and paperwork. Despite some lost earnings from her prior career, she reported low levels of stress and burden and a good quality of life.

P4020 had four children and had been married to the Veteran for 14 years. She had witnessed significant physical (back and shoulder pain) and mental health changes (increased anger and isolation) after his first combat deployment. Like P4004, P4020 assisted with her husband's medical care and engaged in couples counseling. P4020 remained a low-burden case at baseline and 12 months. Despite being the sole support for him outside of the mental health care team, she had a strong social network, including family, friends, and coworkers, and engages in self-care through meditation, traveling, and receiving massages.

## Discussion

In this study, we draw on self-reported caregiver burden in addition to interviews spaced 12 months apart to capture the experiences of CPs of US military Veterans. The objective was to use a mixed methods approach to categorize 36 CPs into discernible groups through a conceptual framework that posits three aspects of burden: multi-faceted strain, self-perception, and change over time. We demonstrated how CPs were categorized as high or low burden initially, but also how some cases shifted categories based on changing circumstances and factors exacerbating or diminishing personal strain. A life course perspective developed for military households can address the unique challenges of managing civilian-military cultural issues, the strain of emotional dysregulation among recently separated Veterans, and intertwined physical and mental health conditions (38, 64). These findings offer longitudinal evidence that supports the broader framework promoted by the Dole Foundation's caregiver journey map (30). In particular, our findings reinforce how the process of self-identifying as a caregiver can be a gradual process, followed by critical points where burden and wellbeing may fluctuate.

Drawing on an existing conceptual model for caregiver burden (35), we closely examine specific cases to identify antecedents and consequences of CP burden that are specific to providing support for military Veterans. Existing studies tend to focus on either the potentially positive aspects of caregiving or emphasize the emotional distress (65) but are less likely to describe financial strain or the opportunity costs of caregiving (66). This sample was notable for its relatively small use of available resources; a single CP was enrolled in a formal caregiver program, and CPs reported little training or preparation for support tasks. CPs in this study described how they supported Veterans who were dealing with emotional dysregulation. Likewise, CPs play an integral role in monitoring how their partners interact with civilians in stressful environments, often shielding them from potentially challenging spaces or repairing conversations where Veterans encountered communication problems with civilians.

In contrast to the preponderance of caregiving studies with older participants, these findings highlight the disruptions that occur for those in the early adult stage (ages 17–45), as they shift careers, manage childcare responsibilities, and transition from military service. CPs discussed balancing support for their partner alongside caring for children and pursuing their own career. Consistent with other studies (67), spousal caregivers reported a lack of confidence in their ability to support their partners and also described difficulties with intimacy. Male spouses in particular reported more issues with role strain—that is, feeling unsure or inadequate in their ability to support their spouse. Multi-faceted strain in the context of the challenges of early adulthood reinforces the importance of a life course perspective (31, 32).

Limitations of these findings should be noted. CPs were recruited based on being nominated by a participating Veteran, which likely limited the heterogeneity of the sample. Most participating Veterans were male, whereas 72% of CPs were female, and most had been caring for the Veterans for more than 5 years. More research is needed to better understand how findings might apply across different sociodemographic groups. The study included multiple data collection points which offered a window into how CPs changed over a 12-month period.

## Conclusion

Few studies have examined how the experiences of unpaid caregivers support military Veterans with invisible injuries such as post-traumatic stress disorder and traumatic brain injuries. Findings suggest the unique needs of individuals who support military Veterans with invisible injuries, highlighting variations and diachronic elements of caregiving. Distinguishing between the experiences of CPs who report high and low burden offers insight into how unpaid caregivers are affected by childcare, financial responsibilities, or incomplete knowledge of appropriate mental health care.

## Data availability statement

The original contributions presented in the study are included in the article/supplementary material, further inquiries can be directed to the corresponding author.

## Ethics statement

The studies involving humans were approved by Indiana University IRB and Roudebush VA Medical Center. The studies were conducted in accordance with the local legislation and institutional requirements. The participants provided their written informed consent to participate in this study.

## Author contributions

NR: Conceptualization, Data curation, Formal analysis, Funding acquisition, Investigation, Methodology, Project administration, Resources, Supervision, Validation, Visualization, Writing – original draft, Writing – review & editing. MF: Conceptualization, Investigation, Resources, Visualization, Writing – original draft, Writing – review & editing, Project administration. AM: Data curation, Formal analysis, Visualization, Writing – original draft, Writing – review & editing. LD: Data curation, Writing – review & editing. A-ND: Writing – review & editing. DN: Writing – review & editing. KS: Writing – review & editing. GT: Funding acquisition, Conceptualization, Writing – review & editing.
